# Deposition of Antibody Modified Upconversion Nanoparticles on Glass by a Laser-Assisted Method to Improve the Performance of Cell Culture

**DOI:** 10.1186/s11671-019-2918-x

**Published:** 2019-03-15

**Authors:** Songlin Yang, Wai Hei Tse, Jin Zhang

**Affiliations:** 1 Department of Chemical and Biochemical Engineering, University of Western Ontario, London, ON N6A 5B9 Canada; 2 Department of Medical Biophysics, University of Western Ontario, London, ON N6A 3K7 Canada

**Keywords:** Cell culture, Laser-assisted deposition, Upconversion nanoparticles, Antibody

## Abstract

A suitable surface is vital for maintaining or even promoting cells’ function and communication. Recently, studies show that nanostructured coatings could have a potential in improving cell adhesion. However, it hardly minimizes the contamination by using traditional solution-coating technology. Matrix-assisted pulsed laser evaporation (MAPLE) technique is a contamination-free process and demonstrates an efficient process to deposit biopolymer without damaging their backbone on the surface of various substrates. Here, upconversion nanoparticles (NaGdF_4_: Yb^3+^, Er^3+^) with/without immunoglobulin G (IgG) modification were produced by a one-pot synthesis method. The average size of the upconversion nanoparticles (UCNPs) is 50 ± 8 nm. IgG bio-conjugated on the surface of UCNPs can be directly observed by transmission electron microscope (TEM). MAPLE system utilizing a Nd:YAG laser (λ = 532 nm, ν = 10 Hz) is applied to deposit UCNPs with/without IgG modification on the glass bottom of culture dish. In addition, the behaviors of human umbilical vein endothelial cells (HUVECs) cultured on the culture dishes coated with UCNPs with/without IgG have been studied as compared to the control sample, glass coated with gelatin. No toxic effect is imposed on cells. The results of this work indicate that the deposition of UCNPs with/without antibody by the MAPLE technique could enhance the adhesion and proliferation of cells.

**Electronic supplementary material:**

The online version of this article (10.1186/s11671-019-2918-x) contains supplementary material, which is available to authorized users.

## Introduction

Epithelial cells can be found at the inner and outer surfaces of the human body, including the skin, intestines, airway, and reproductive tract. Epithelial cells not only provides a safety shell against the dirt and microbes, but they also exhibit important functions, e.g., stretch, tracks, etc. [1]. Therefore, epithelial cells have been extensively used in tissue engineering and tissue regeneration. The interaction between epithelial cells and the surface of substrates is vital for maintaining cells’ function and communication. Normally, a protein-based coating, e.g., rat-tail collagen, is applied to allow the epithelial cells growing on the petri dish, or glass, for further studies. Recently, nanomaterials coated on a substrate demonstrate the potential for the control of the growth of cells by utilizing the fine morphologies, special textures/patterns of the nanostructured coating [2–4]. In addition, luminescent nanomaterials have shown the significant advantages over traditional organic dye in studying the interaction of cell-cell, and cell-surface because of their highly stable photoluminescence properties. It is interesting to find out the interaction of cells and a surface coated with protein-modified luminescent nanostructures.

The upconversion phenomenon, first investigated in 1959, is the sequential absorption of two or more photons to emit a light with high energy [5, 6]. The lanthanide-doped upconversion nanoparticles (UCNPs) consists of three different components including activator, sensitizer, and host matrix. The lanthanide ions such as Er^3+^, Ho^3+^, and Tm^3+^ could play a role as activators since they possess unique energy-level structures [7–11]. Yb^3+^ ion is the most common sensitizer which can be applied to transfer the energy from excited light to the activators [12–14]. Both oxidic materials and fluoride materials are normally used as the crystal host [15–17]. Upconversion nanoparticles, emitting light from the visible range to the near-infrared range under the excitation of the near-infrared (NIR) light, can be applied in deep tissue bioimaging because of the lower scattering coefficient of NIR light known as the “therapeutic window” [18]. Recently, various surface modification of UCNPs have been developed for biological labeling/sensing [19, 20]. For instance, avidin was conjugated onto hexanedioic acid (HAD) modified on the surface of UCNPs to demonstrate the interaction with antibodies [21]. ssDNA-modified core-shell UCNPs are developed for detecting specific oligonucleotides [24].

On the other hand, immunoglobulin G (IgG), an antibody found in blood and extracellular fluid, controls the infection of tissue. The interactions between IgG and nanoparticles have been studied, for instance, IgG can be used as a template to produce gold nanoparticles, and IgG modified magnetic nanoparticles to label bacterial cells [22, 23]. However, only few studies have been reported on modifying IgG onto UCNPs for cell culture or tissue culture.

Conventional methods such as sol-gel methods, spin coating, and solvent evaporation have been applied in deposit biomolecules modified nanoparticles on a substrate for biomedical assay [27, 28]. However, solution-coating methods for deposition of proteins or protein-based nanostructures hardly minimize the contamination. Laser deposition exhibits well-controlled thickness and avoids the operation contamination occurring in chemical depositions. Very few laser deposition techniques have been used for developing a protein-based surface for cell culture.

Unlike conventional physical deposition methods, matrix-assisted pulsed laser evaporation (MAPLE) technique does not directly ablate the target materials; instead, most energy of laser is absorbed by the frozen solvent (matrix) [24, 25]. In a MAPLE process, target materials dispersed in a highly volatile solvent (matrix) are introduced into the target holder cooled with liquid nitrogen [26–30]. Under the laser irradiation, the target materials can be transported to the substrate with evaporating solvent. APLE technique has been applied in various fields including sensors, organic electronic devices, drug delivery, implants coating, etc. [31–35]. However, very few work has been reported on the effect of the substrate with MAPLE-deposited biomolecule/protein nanoparticles on the performance of cell culture.

In this study, we produced UCNPs (NaGdF_4_: Yb^3+^, Er^3+^) and immunoglobulin G (IgG)-modified UCNPs (UCNPs-IgG) by hydrothermal method. Following that, UCNPs with/without modification of IgG were directly deposited on the glass bottom of a cell culture dish by using a MAPLE process with 532 nm Nd:YAG laser. The human umbilical vein endothelial cells (HUVECs) were seeded on the glass deposited with UCNPs to investigate the cytotoxicity of UCNPs’ coating, and the cells’ behaviors on the surface treated with UCNPs.

## Methods/Experimental

### Materials

Gadolinium (III) nitrate hexahydrate (Gd (NO_3_)_3_·6H_2_O, crystals and lumps, 99.9% trace metals basis), ytterbium (III) nitrate pentahydrate (Yb (NO_3_)_3_·5H_2_O, 99.9% trace metals basis), erbium (III) nitrate pentahydrate (Er (NO_3_)_3_·5H_2_O, 99.9% trace metals basis), sodium fluoride (NaF, BioReagent, suitable for insect cell culture, ≥ 99%), branched polyethylenimine (PEI, average Mw ~ 800 by LS, average Mn ~ 600 by GPC), anti-human IgG (Fab specific)–FITC antibody produced in goat (affinity isolated antibody, buffered aqueous solution), 4′,6-diamidine-2′-phenylindole dihydrochloride (DAPI), and Phalloidin–Tetramethylrhodamine B isothiocyanate (Phalloidin-TRITC) were purchased from Sigma-Aldrich. Ethylene glycol (EG) was purchased from Fisher chemical. Isopropanol (2-propanol) was purchased from Caledon laboratory chemicals.

### Synthesis of UCNPs With and Without IgG by Using a One-Pot Process

The UCNPs (NaGdF_4_: Yb^3+^, Er^3+^) were synthesized by a modified one-pot method [36]. Briefly, 720 mg of Gd (NO_3_)_3_·6H_2_O, 170 mg of Er (NO_3_)_3_·5H_2_O, 160 mg of Yb (NO_3_)_3_·5H_2_O, and 20 ml ethylene glycol were added into a three-neck flask. Then, 0.7 g of PEI was gently added to the flask. Then, 336 mg of NaF dissolved in 10 ml ethylene glycol was added dropwise to the flask. The mixture was heated to 200 °C and refluxed for 6 h in nitrogen protection. The reaction products were collected by centrifugation and washed several times with ethanol/distilled water. The product was dried at 60 °C.

**Fig. 1:**
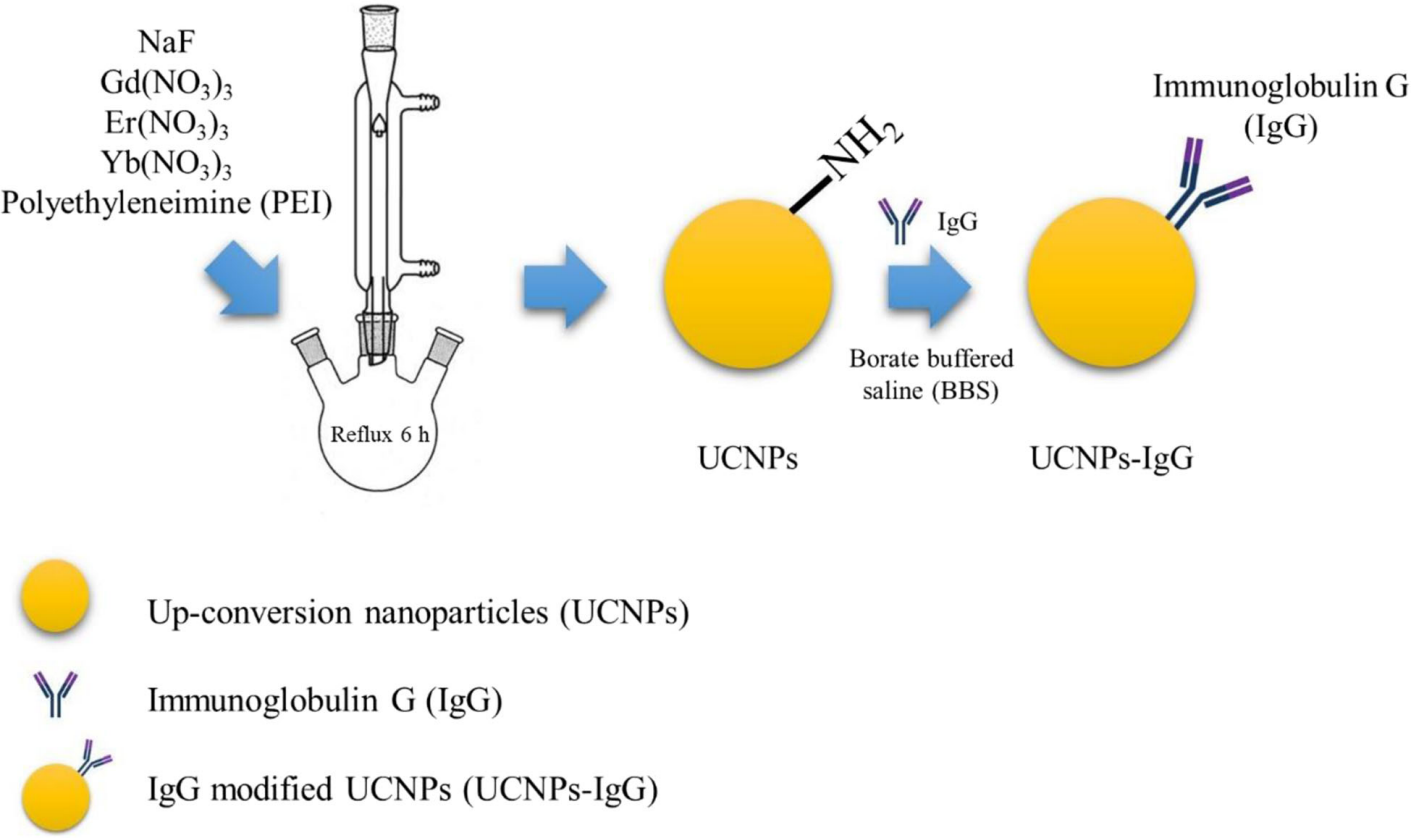
Schematic of one-pot method for producing UCNPs and UCNPs-IgG

IgG antibodies were modified on the surface of UCNPs by amide linkages. As shown in Fig. 1, 15 mg of UCNPs powder were dissolved in 15 ml distilled water. Then, 5 ml of borate-buffered saline (BBS, pH = 8) and 20 μg of IgG were added to the solution. The solution was stirred at room temperature for 2 h. The product was washed and collected by centrifugation.

### Deposition of UCNPs With/Without IgG by MAPLE

**Fig. 2:**
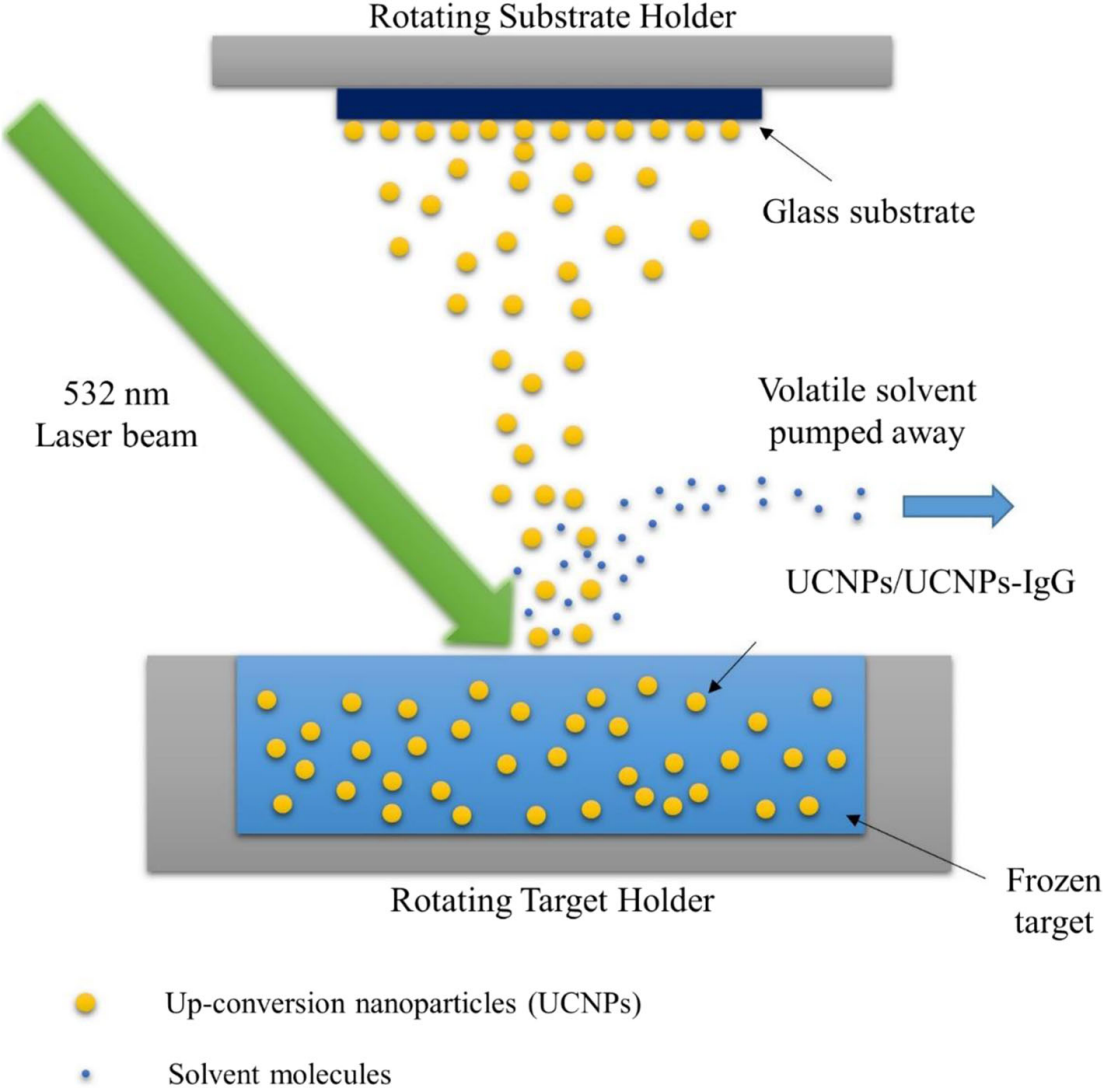
Schematic of deposition of UCNPs and UCNPs-IgG by using MAPLE technique

The laser deposition process was performed by MAPLE 2000 equipment (project 206, PVD Products, Inc., USA) affiliated with a 532-nm Nd:YAG laser. The deposition process is shown in Fig. 2. Target nanoparticles were dissolved in isopropanol with a concentration of 1 wt.%. The mixture was frozen in the target holder cooled by liquid nitrogen. No other additional additives and surfactants were used in this process.

Further, 532 nm Nd:YAG laser was applied with laser frequency at 10 Hz and the τ_fwhm_ ≅ 200 μs. Laser spot area size was 0.63 cm^2^ and laser fluence was 150 mJ/cm^2^. The glass substrates were treated with a 2 wt% solution of gelatin which was fixed on the substrate holder and the temperature of substrates was 25 °C during the whole experiment process. The laser deposition process was conducted under 1 × 10^−6^ Torr. As per our previous studies [42, 43], the deposition time was set to 2 h. The substrate-to-target distance was 4.5 cm (vertical configuration). The target holder and the substrate holder were rotating (target: 10 rpm, substrate: 25 rpm) during the laser irradiation period.

### Materials Characterizations

UCNPs and UCNPs-IgG before the MAPLE deposition were characterized by transmission electron microscope (TEM, Philips CM-10, operating at 80 kV). Fourier-transform infrared spectra (FTIR) of the UCNPs and UCNPs-IgG were obtained by a Bruker Vector 22 FTIR spectrometer (scan range 600 cm^−1^~4500 cm^−1^, resolution 4 cm^−1^, 64 scans). The photoluminescence properties of UCNPs were studied by using QuantaMaster™ 40 Spectrofluorometer (Horiba Canada—Photon Technology International Inc.). The growth of human umbilical vein endothelial cells (HUVECs) on the glass surface with/without the depositions of nanoparticles was investigated by a confocal microscope (Zeiss LSM 5 Duo Vario Microscope). Hitachi S-3400 N scanning electron microscope attached with INCA PentaFET-x3 EDX system (Oxford Instruments) was applied to perform the energy-dispersive X-ray spectroscopy (EDX).

### Study on Cell Behaviors

Human umbilical vein endothelial cells (HUVECs, American Type Culture Collection) were applied to study the biocompatibility of UCNPs with/without IgG modification after the laser deposition treatment. HUVECs were seeded on the glass surface deposited with UCNPs and UCNPs-IgG. The glass substrate treated with 2 wt.% solution of gelatin (without UCNPs) is used as the control after the MAPLE deposition treatment. The deposited samples were soaked in MCDB medium (10% fetal bovine serum, 1% penicillin, and amphotericin B) for the seeding of HUVECs. The HUVECs were cultured at 37 °C for 24 h. HUVECs were fixed on substrates’ surface with 4% formalin for 2 h for obtaining the confocal imaging (Zeiss LSM 510 Duo). The cells were stained with Phalloidin-TRITC and DAPI. The samples were washed with PBS (pH 7.4) and treated with the anti-fade agent.

### Study on Cytotoxicity

The HUVECs were cultured in MCDB medium (10% fetal bovine serum, 1% penicillin, and amphotericin B) on the surface of the deposited samples. After transfer to culture dishes, the cells were cultured for 24 h (37 °C, 5% CO_2_) on the surface of the samples. Approximately 100,000 cells were seeded on the surface of each sample (with/without UNCPs deposition). All samples including the control, the glass substrates with the deposition of, UCNPs, and UCNPs-IgG were measured in triplicate. Then the MTT agent (3-(4, 5-dimethyl thiazolyl-2)-2, 5-diphenyl tetrazolium bromide) was added to the cell media and cells were incubated for 3 h. The cell media was removed and the wells were rinsed two times with PBS. Then DMSO was added to each well to dissolve the formazan. The liquid was transferred to the 96-well plates and analyzed with the bio-kinetic reader at 490 nm (Bio-Tek Instruments EL340I Microplate Reader).

## Results and Discussion

### Characterization of UCNPs/UCNPs-IgG Before MAPLE Deposition

**Fig. 3:**
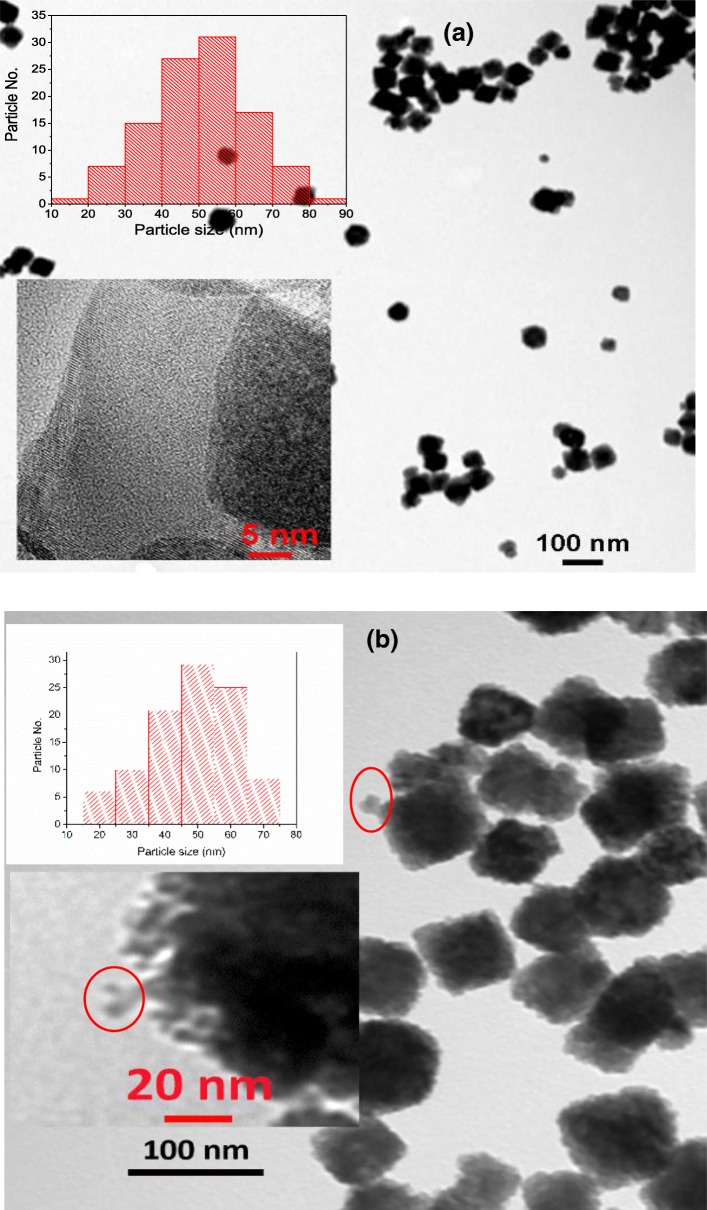
TEM micrographs of **a** UCNPs and **b** UCNPs-IgG before MAPLE deposition

IgG antibodies were conjugated onto amine-modified UCNPs through amide linkages. The UCNPs with/without IgG were characterized by TEM. Figure 3a is the TEM micrograph of cubic UCNPs. The average size of the UCNPs is estimated at 50 ± 8 nm. The small inset of Fig. 3a is the micrograph of high-resolution TEM (HRTEM). UCNPs have highly crystalline structures. The measured interplanar distance between two adjacent lattice planes was 0.312 nm, corresponding to a (1 1 1) plane of cubic phase NaGdF_4_ (JCPDS 27-0697). Figure 3b is the TEM micrograph of UCNPs conjugated with IgG (UCNPs-IgG). The UCNPs modified with antibodies remained the cubic shape, and the average particle size is around 54 ± 8 nm. The IgG antibodies bioconjugated onto UCNPs can be observed directly by TEM as shown in Fig. 3b, marked with red circle. The size of the IgG antibodies is around 10 ± 5 nm which is similar to the theoretical calculation and experiment measures [44, 45].

**Fig. 4:**
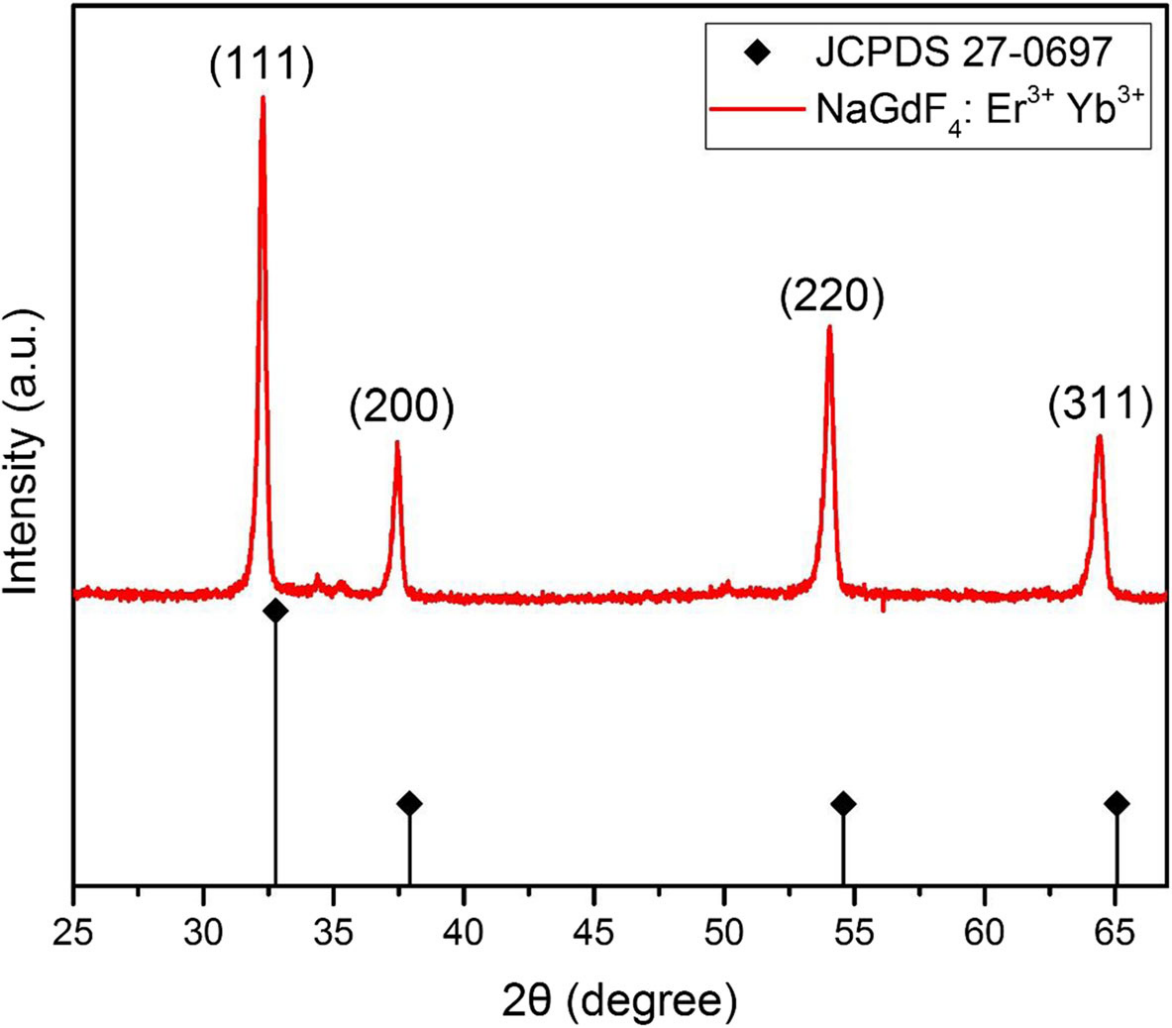
XRD Profile of NaGdF_4_: Er^3+^, Yb^3+^ upconversion nanoparticles

The crystal structure of UCNPs (NaGdF_4_: Yb^3+^, Er^3+^) was revealed by X-ray powder diffraction (XRD) pattern as shown in Fig. 4. Four peaks at 32°, 37°, 54°, and 65° in XRD profile are attributed to (111), (200), (220), and (311) crystal planes, which is the same with the standard XRD pattern of cubic phase NaGdF_4_ (JCPDS 27-0697) and references [36, 37]. Both HRTEM and XRD measures confirm the UCNPs’ crystal structure.

**Fig. 5:**
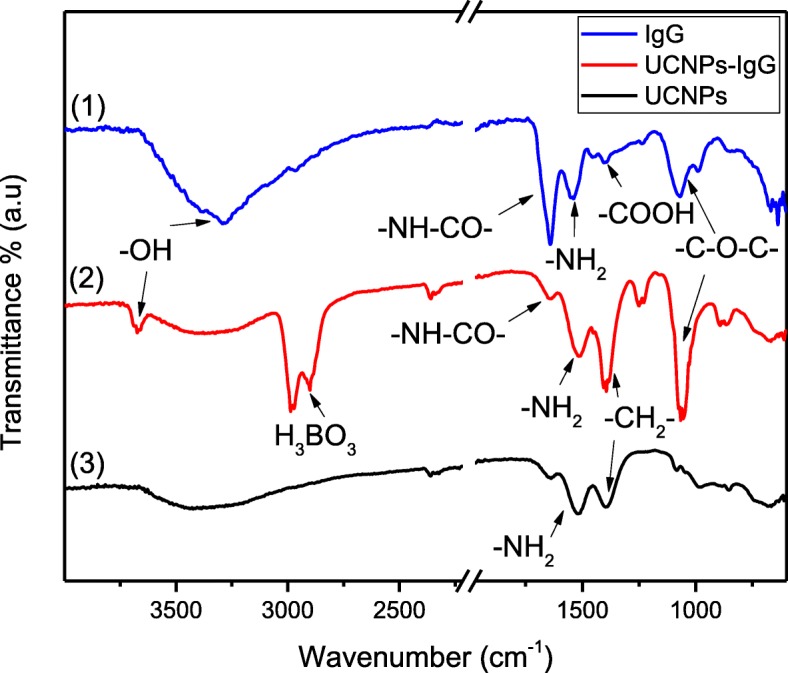
FTIR spectra of UCNPs-IgG and UCNPs, respectively, made by one-pot process

To further investigate the bioconjugation of IgG onto UCNPs, Fourier-transform infrared spectroscopy (FTIR) was employed. As shown in Fig. 5, the bending vibration bands of amine groups is observed at 1515 cm^−1^ and 1511 cm^−1^ in the spectra of UCNPs and UCNPs-IgG since both the PEI and IgG antibodies possess amino groups. The peaks at 2987 cm^−1^, 2900 cm^−1^, and 1400 cm^−1^ can be attributed to the stretching vibrations of -CH_2_- and C-C bonds, respectively. The stretching vibration band of the hydroxyl group (-OH) is observed at 3673 cm^−1^ in the spectrum of UCNPs-IgG due to the carboxyl group of IgG. The peak at 1249 cm^−1^ and 1650 cm^−1^ are attributed to the stretching vibration bands of the carbon-oxygen bond and amide linkage, respectively, in the spectrum of the IgG-modified UCNPs. These peaks reveal the presence of the IgG antibodies on the surface of the UCNPs-IgG.

### Characterization of UCNPs With/Without IgG After MAPLE Deposition

**Fig. 6:**
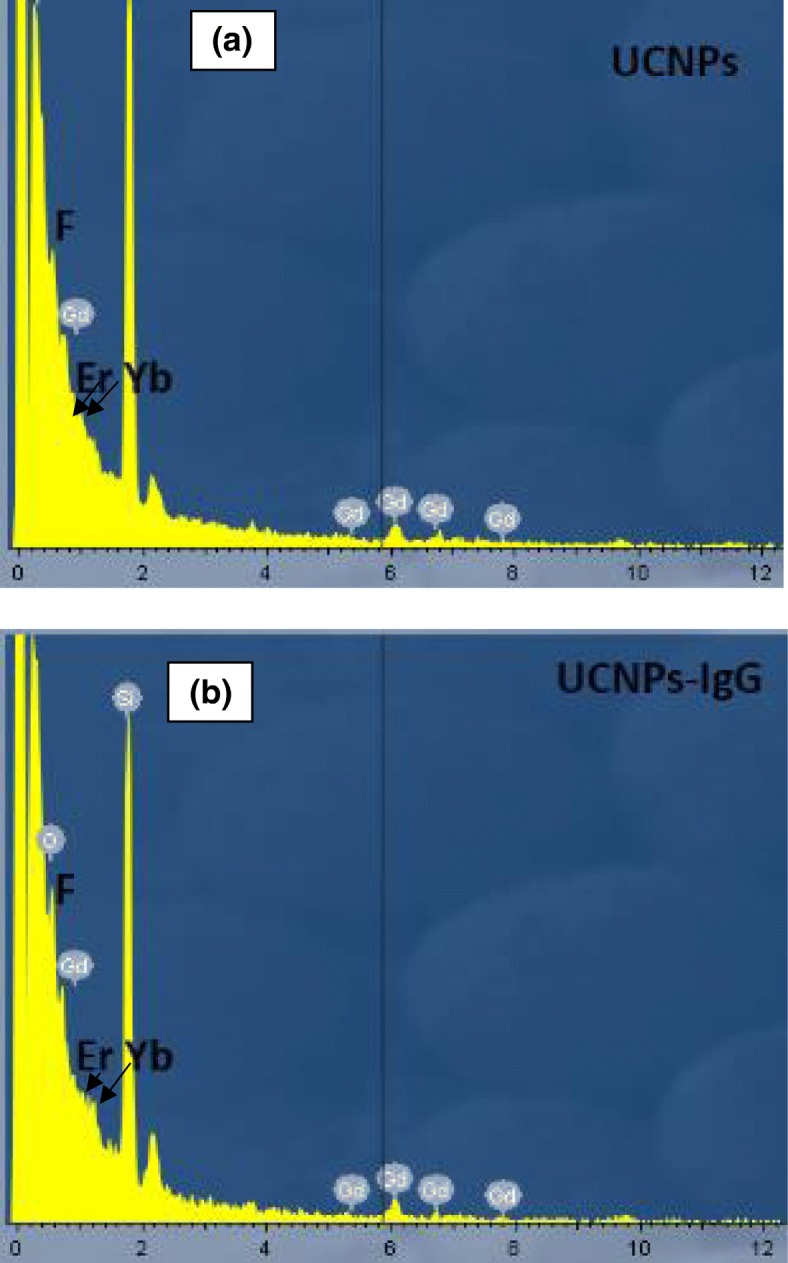
EDX spectra of **a** samples after MAPLE treatment and **b** samples without MAPLE treatment (bare glass)

UCNPs and UCNPs-IgG were deposited by MAPLE process on cell culture dishes with the glass bottom. The surface of glass before and after the deposition of UCNPs and UCNPs-IgG, respectively, were characterized by the energy-dispersive X-ray spectroscopy (EDX). Figure 6 shows the presence of elements of gadolinium (Gd), erbium (Er), ytterbium (Yb), and fluorine (F) in samples of glass coated with UCNPs (Fig. 6a) and UCNPs-IgG (Fig. 6b), respectively. In addition, the photoluminescence of UCNPs and UCNPs-IgG after the MAPLE deposition with longer irradiation time were measured. Both green (540 nm) and red (650 nm) emissions can be observed under the excitation of 980 nm as shown in Additional file 1: Figure S1 in the supporting file. It is noted that the intensity of the emission is slightly different between samples of UCNPs with and without IgG, which could be caused by the surface defects, or the measure error. Further study will be conducted. Consequently, the MAPLE deposition can maintain the structure and properties of UCNPs with/without IgG.

**Fig. 7:**
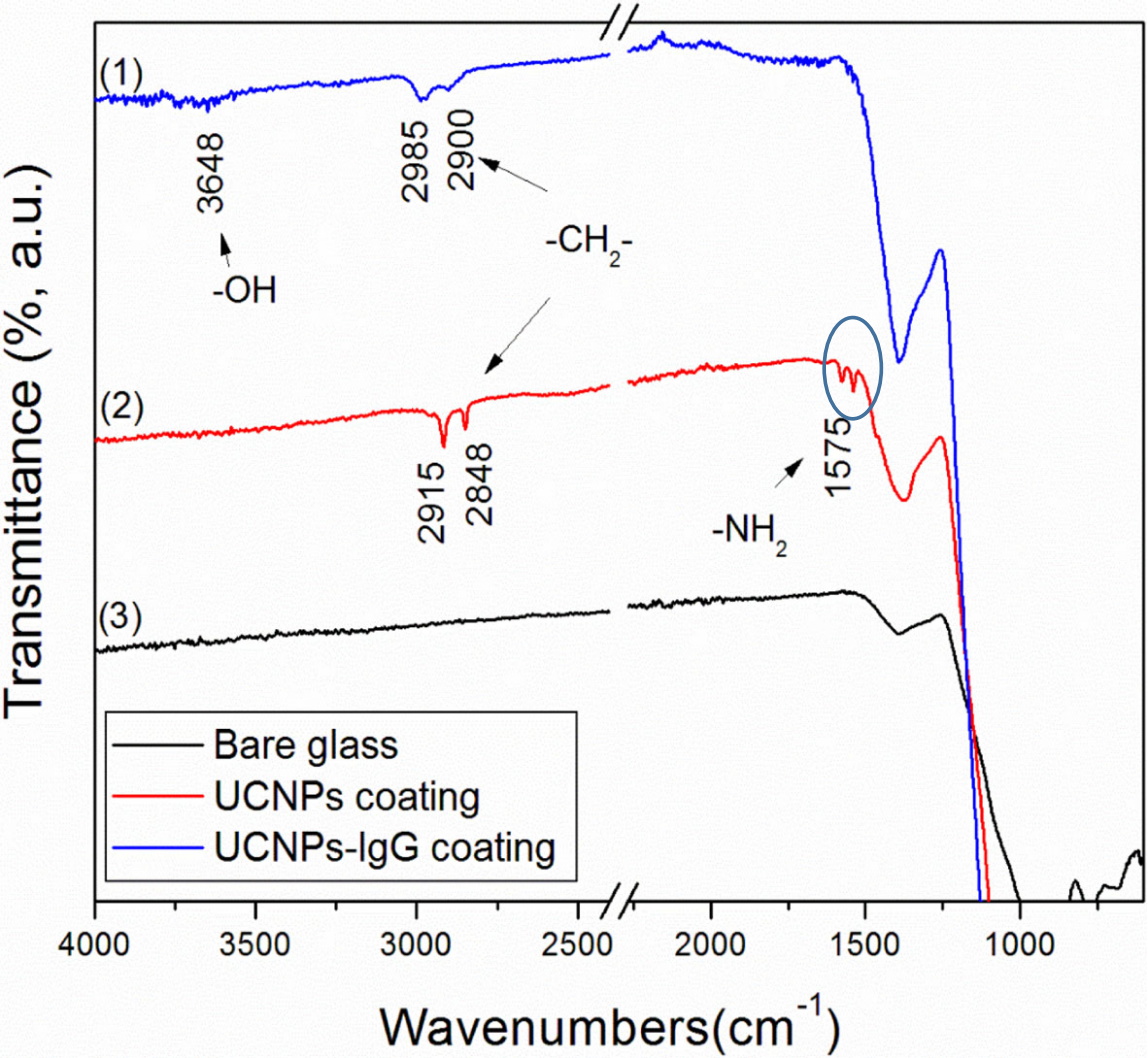
FTIR spectra of bare glass and glass coated with UCNPs-IgG and UCNPs, respectively

FTIR was used to investigate UCNPS and UCNPs-IgG coatings made by MAPLE technique in comparison with the bare glass sample. The FTIR spectra of the samples deposited with UCNPs, UCNPs-IgG by the MAPLE deposition, and blank sample (bare glass substrate) are displayed in Fig. 7. The stretching vibration bands of –OH groups at 3648 cm^−1^ is attributed to carboxyl group of IgG, which is only observed in spectrum-1, i.e., UCNPs-IgG coating. This result indicates the existence of the UCNP-IgG on substrates’ surface. In spectrum-2 (UCNPs coating), the bending vibration band at 1575 cm^−1^ is attributed to the amine group modified on the UCNPs, while the bending vibration band of amine group is hardly observed in spectrum-1 (UCNPs-IgG) because of the successful bioconjugation. Consequently, MAPLE technique is able to maintain the properties and microstructures of UCNPs modified with antibodies.

Compared to the spectrum of the bare glass (spectrum-3), the stretching vibration bands of methylene groups are around 2985 cm^−1^ and 2900 cm^−1^ in both spectrum-1 and spectrum-2 due to the carbon-based functional groups on the surface of UCNPs. It is noted that the peaks of coatings in Fig. 7 are significantly weaker than the peaks in Fig. 5 which are caused by the low quantities of nanoparticles deposited on the substrate surface, and the changes of spectra at 1250 cm^−1^ in Fig. 7 as compared to Fig. 5 stems from the effect of glass substrate.

### Cell Behaviors on the Different Coatings

**Fig. 8:**
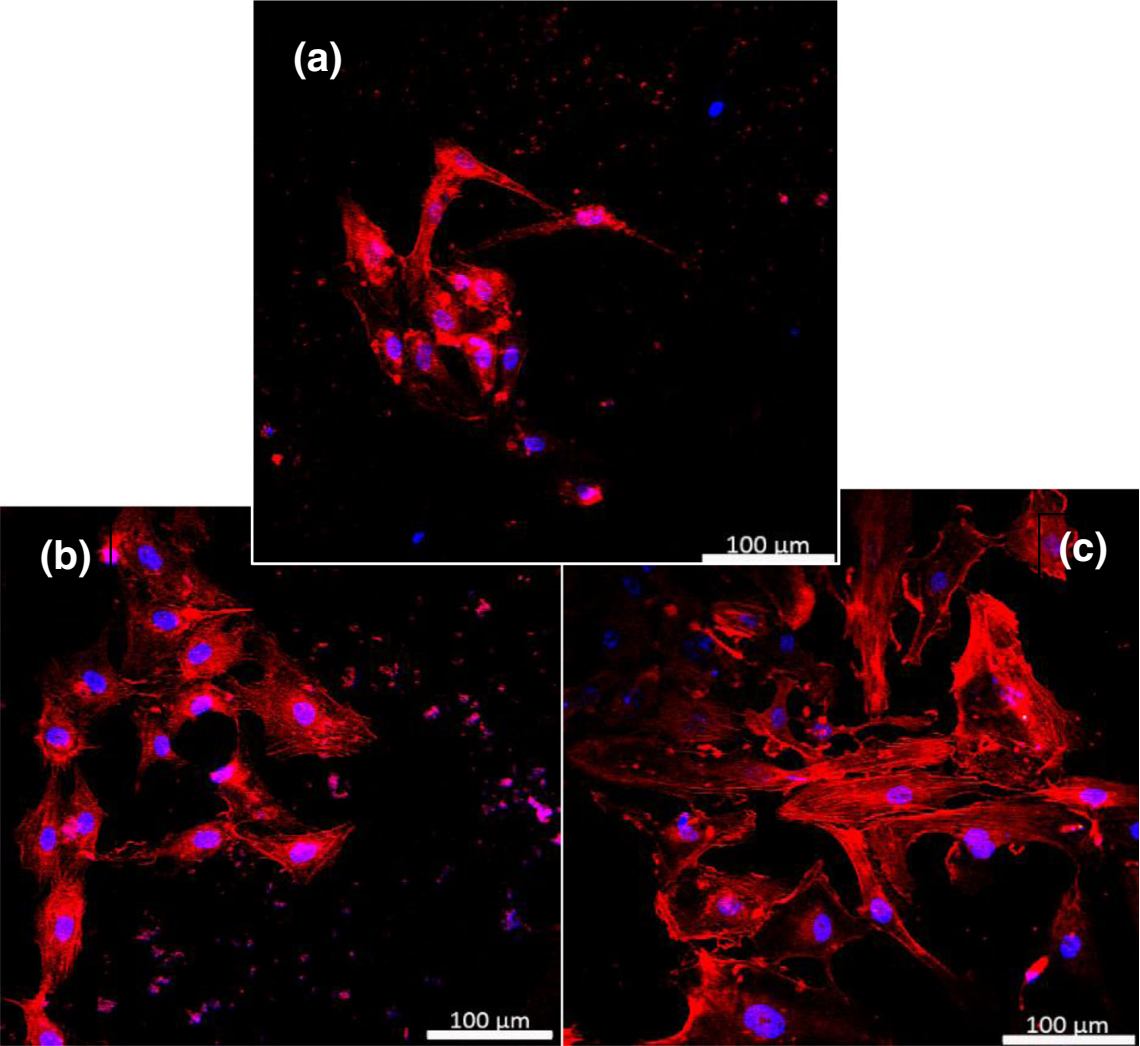
Confocal micrographs of HUVECs on the surface of **a** control, **b** UCNPs, and **c** IgG-modified UCNPs

Traditional methods for HUVEC cells require coating a protein layer which is used as our control in the study of the cell culture performance. Therefore, three different coatings in this study are (1) control (glass coated with gelatin), (2) glass coated with UCNPs, and (3) glass coated with UCNPs-IgG. Figure 8 presents the confocal microscope micrographs of cells cultured on different surfaces for 24 h. The amounts of HUVECs growing on the surface coated with UCNPs and UCNPs-IgG, respectively, are similar with that of control. However, the behaviors of HUVEC cells growing on the surfaces modified with UCNPs-IgG are improved dramatically in the first 24-h culture period. The cells growing behaviors within the culture period included the area of the cells, the length of connections, the length of cells, and total cell number on the samples’ surface were investigated and analyzed.

**Fig. 9:**
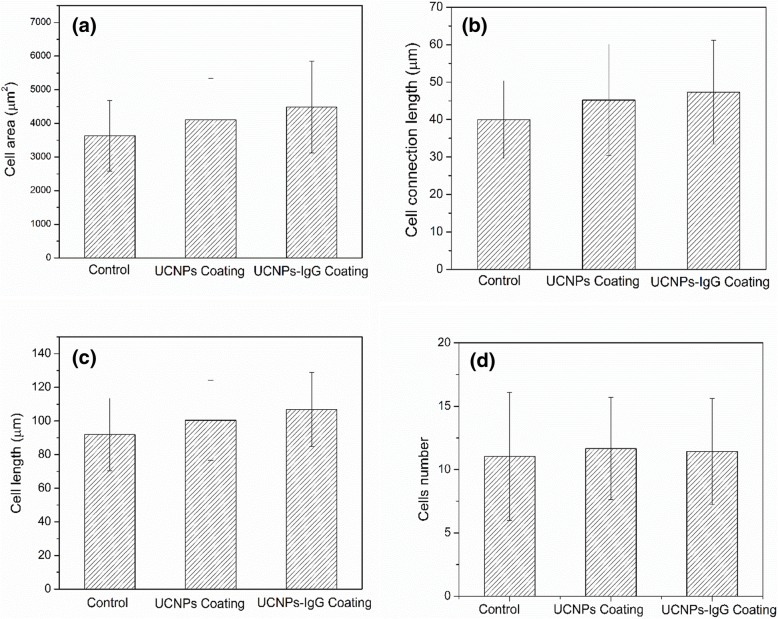
HUVECs cultured on different coatings including control, UCNPs, UCNPs-IgG: **a** cell area, **b** cell connection length, **c** cell length, and **d** cells number

Figure 9 displays the statistical analysis of the cell behaviors after 24-h culturing on the three different surfaces. The area of the cells (Fig. 9a), the length of connections (Fig. 9b), the length of cells (Fig. 9c), and cells number (Fig. 9d) were investigated by the ImageJ software. The lengths of the cells are defined as the largest length value of this cell. The length of the connection is defined as the distance between the edge of the cell’s tips and the cell nuclei. Compared to that of cells growing on control sample, the cell area of HUVEC cells on the surface modified with UCNPs and UCNPs-IgG increases with 11.2% and 22.2%, respectively; the length of connections of HUVEC cells on the surface modified with UCNPs and UCNPs-IgG increases with 12.5% and 17.5%; and the length of HUVEC cells on the surface modified with UCNPs and UCNPs-IgG increases with 8.2% and 17.3%. Moreover, the analyzed results of cells number show that the amount of the cells on the surface of the UCNPs-based sample is about 8% higher than that of control. These results indicate that both UCNPs samples and UCNPs-IgG samples are biocompatible with the HUVECs cells which are consistent with the previous works [38]. The growth of HUVEC cells cultured on the surface deposited with UCNPs and UCNPs-IgG has been promoted in terms of cell area, cell length, and the length of connection. Previous studies indicate that nanostructures could trigger endothelial activation of HUVEC cells [2–4, 39]. It has been reported that releasing of inflammatory mediators and upregulation of adhesion molecules of HUVECs would be observed when exposed to a variety of nanoparticles [40]. Inflammatory mediators could promote the angiogenesis and pro-angiogenesis effects based on the previous studies [2, 41]. In addition, recent work indicates that IgG could promote the angiogenesis-like transformation of HUVECs [3].

### Effect of the Coating of UCNPs With/Without IgG on Cell Viability

**Fig. 10:**
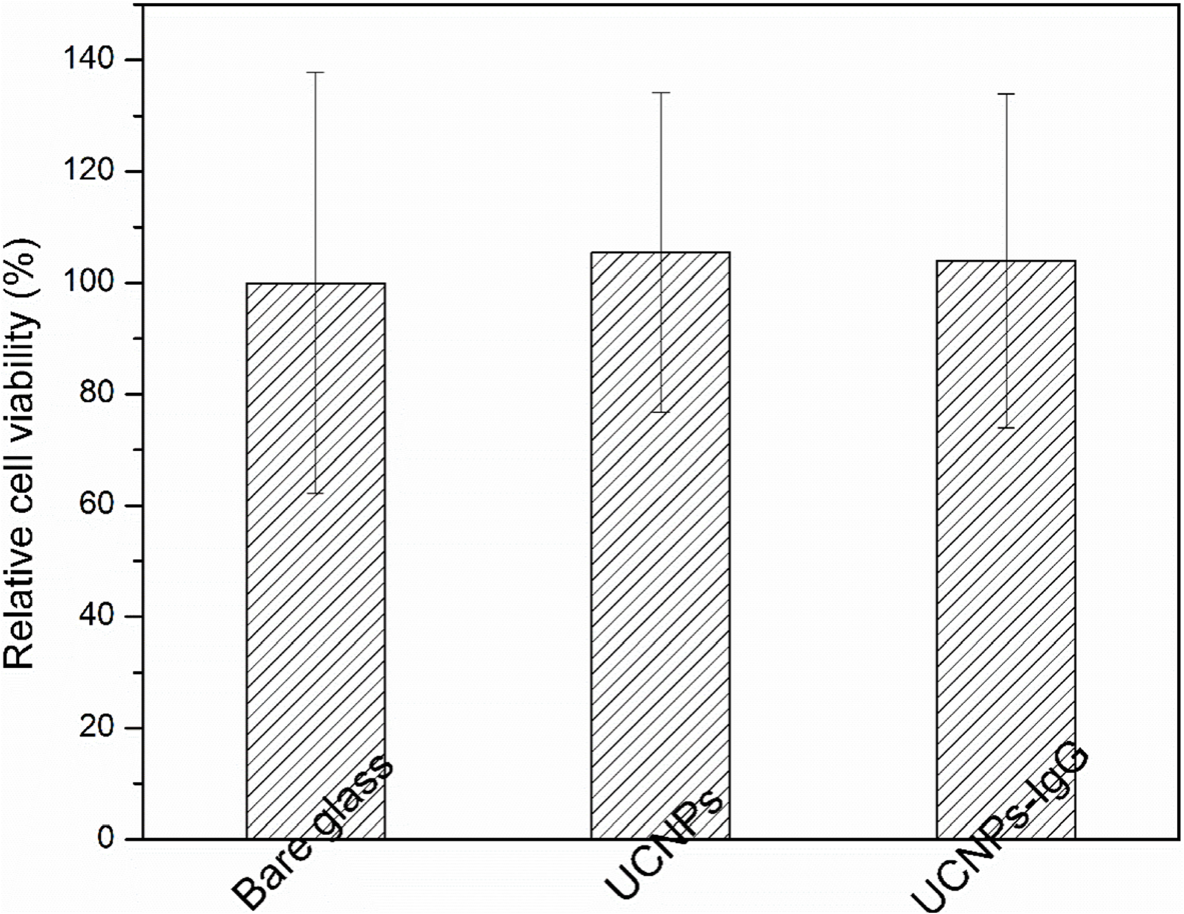
Cell viability of HUVEC cells growing on bare glass, coatings with UCNPs and UCNPs-IgG, respectively, and glass coated with gelatin used as control

The cell viability was studied by using HUVEC cells cultured on the different surfaces. Figure 10 shows the relative cell viability on different surfaces: control (glass coated with gelatin), bare glass, UCNPs deposited on glass substrates, and UCNPs-IgG deposited on glass substrates. The relative cell viability of the bare glass, glass coated with UCNPs, and glass coated with UCNPs-IgG were 99.6%, 105.44%, and 103.96%, respectively. Some studies show that PEI with high concentration may have toxic effect, particularly when applied at high concentration, but the use of PEI as a ligand is acceptable and shows negligible cytotoxicity [44–47]. Due to the mechanism of MAPLE, the amount of the UCNPs/UCNPs-IgG on the substrate surface is much lower than the threshold (1 mg/ml). Clearly, the deposition of UCNPs and UCNPs-IgG on the glass by MAPLE does not impose toxic effects on the HUVEC cells. It is noted that biocompatible materials normally can have a relative cell viability (> 85%) of cells. [8]

## Conclusions

In summary, the UCNPs and UCNPs-IgG were successfully synthesized by the one-pot method and have been deposited on culture dishes with glass bottom by MAPLE technique. The results of TEM and FTIR reveal the successful bioconjugation of IgG on the surface of UCNPs. The average particle size of the UCNPs is 50 ± 8 nm. The UCNPs modified with antibodies remained the cubic shape, and the average particle size is around 54 ± 8 nm. The bioconjugation of IgG on UCNPs can be observed directly by TEM which has the average size of 10 ± 5 nm. The FTIR spectra also confirmed the presence of the carboxyl group/peptide bond of the antibody modified on the surface of UCNPs. MAPLE deposition process was used to deposit UCNPs and UCNPs-IgG on glass substrate. The results of EDX, FTIR, and PL measures indicate the retention of structures and properties of UCNPs and UCNPS after MAPLE deposition. Our study demonstrates that the MAPLE process can achieve the retention of the properties and structures of UCNP with/without the modification of antibody. In addition, the performance of cell culture has been statistically studied by culturing HUVEC cell line on the different surfaces treated with UCNPs and UCNPs-IgG, respectively, made by MAPLE process. The cell area, cell length, and the length of connection are very important to support an ideal confluence and the formation of microvessel structures. The glass surfaces treated with UCNPs and UCNPs-IgG samples by MAPLE technique show no toxic effect to the HUVEC cell line. It is expected that MAPLE deposition of UCNPs and UCNPs-IgG could be applied in the fabrication of the new biological devices for tissue engineering and tissue regeneration.

## Additional file

Additional file 1: **Figure S1.** Photoluminescence of UCNPs and UCNPs-IgG deposited on glass by using MAPLE technique. (PDF 144 kb)

## Abbreviations

DAPI: 4′,6-Diamidine-2′-phenylindole dihydrochloride
EG: Ethylene glycol
HUVEC: Human umbilical vein endothelial cells
IgG: Immunoglobulin G
MAPLE: Matrix-assisted pulsed laser evaporation
PEI: Polyethylenimine
Phalloidin-TRITC: Phalloidin–Tetramethylrhodamine B isothiocyanate
UCNPs: Upconversion nanoparticles
