# Supplementary material for: Deposition of Antibody Modified Upconversion Nanoparticles on Glass by a Laser-Assisted Method to Improve the Performance of Cell Culture
Source: Nanoscale Res Lett. 2019 Mar 15;14:101. doi: 10.1186/s11671-019-2918-x (PMC6420592; doi:10.1186/s11671-019-2918-x)
Supplement: Supplementary file 1 — Figure S1. Photoluminescence of UCNPs and UCNPs-IgG deposited on glass by using MAPLE technique. (PDF 144 kb) [file 11671_2019_2918_MOESM1_ESM.pdf]

Supporting Information

Deposition of Antibody Modified Up-conversion Nanoparticles on Glass by a Laser-assisted Method to Improve the Performance of Cell Culture

Songlin Yang, [1] Wai Hei Tse,[2] and Jin Zhang [1,2]*

[1] Department of Chemical and Biochemical Engineering, University of Western Ontario, London, Ontario, Canada N6A 5B9

[2] Department of Medical Biophysics, University of Western Ontario, London, Ontario, Canada N6A 3K7

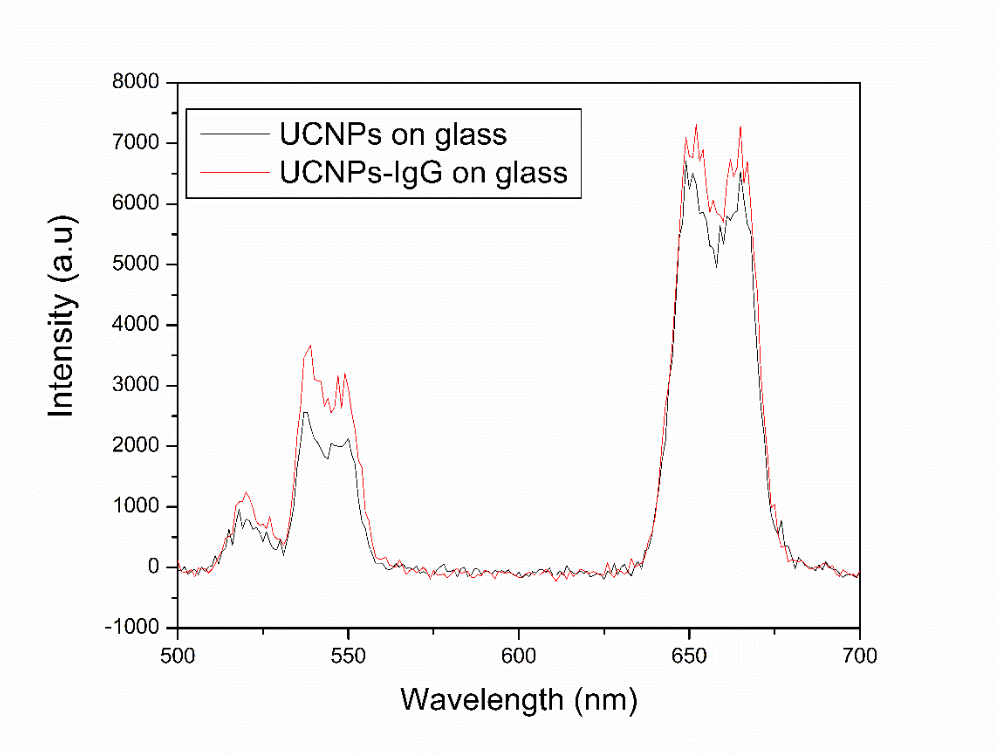

Figure S1. Photoluminescence of UCNPs and UCNPs-IgG deposited on glass by using MAPLE technique.
